# Supplementary material for: Avatar-Based Strategies for Breast Cancer Patients: A Systematic Review
Source: Cancers (Basel). 2023 Aug 9;15(16):4031. doi: 10.3390/cancers15164031 (PMC10452070; doi:10.3390/cancers15164031)
Supplement: Supplementary file 1 [file cancers-15-04031-s001.zip › cancers-2501174-supplementary file S1.pdf]

Supplementary file S1: Study protocol

Adopted from PRISMA-P (Preferred Reporting Items for Systematic review and Meta-Analysis Protocols) and PROSPERO.

**Topic**	**Content**
Title	Avatar-based Strategies for Breast Cancer Patients: A Systematic Review.
Authors	Irene Rodriguez-Guidonet, Paula Andrade-Pino, Carlos Monfort-Vinuesa, and Esther Rincon.
Review team members and their organizational affiliations	• Irene Rodriguez-Guidonet, MA.<br>1. Psycho-Technology Lab (Universidad San Pablo-CEU, CEU Universities).<br>• Paula Andrade-Pino, MA.<br>1. Psycho-Technology Lab (Universidad San Pablo-CEU, CEU Universities).<br>• Prof. Carlos Monfort-Vinuesa.<br>1. Psycho-Technology Lab (Universidad San Pablo-CEU, CEU Universities).<br>2. Departamento de Psicología y Pedagogía, Facultad de Medicina, Universidad San Pablo-CEU, CEU Universities.<br>3. Departamento de Medicina Interna, HM Hospitales, Universidad San Pablo-CEU, CEU Universities, Urbanización Montepríncipe, Email: carlos.monfortvinuesa@ceu.es<br>• Prof. Dr. Esther Rincon<br>1. Psycho-Technology Lab (Universidad San Pablo-CEU, CEU Universities).<br>2. Departamento de Psicología y Pedagogía, Facultad de Medicina, Universidad San Pablo-CEU, CEU Universities.

Contact details for of corresponding author	Esther Rincon, PhD.<br>Psycho-Technology Lab (Universidad San Pablo-CEU).<br>Departamento de Psicología y Pedagogía, Facultad de Medicina, Universidad San Pablo-CEU, CEU Universities, Urbanización Montepríncipe,<br>28660 Boadilla del Monte, España.<br>Phone: +34 913 724 700; ext 15076<br>Email: maria.rinconfernande@ceu.es
Organizational affiliation of the review	Psycho-Technology Lab<br>(Universidad San Pablo-CEU, CEU Universities),<br>Madrid (Spain).
Type and method of review	Systematic review
Contributions	E.R. led the conception and design of the study, the screening of included studies interpretation, and wrote the first draft of the manuscript. I.R.-G. and P.A.-P. were responsible for data extraction, data analysis, and interpretation. I.R.-G., P.A.-P., C.M.-V. and E.R. substantially contributed to analysis, data interpretation, and revised the work critically. All authors gave the final approval of the version to be published and agreed to be accountable for all aspects of the work by ensuring that questions related to the accuracy or integrity of any part of the work are appropriately investigated and re-solved.
Sources/Sponsors	This research received funding for its publication from Universidad San Pablo-CEU, CEU Universities.
Conflict of interest	Authors declare no conflict of interest.
Rationale	In this systematic literature review, we seek to answer the following 2 questions:<br>- (1) Are avatar-based strategies useful to improve QoL and psychological well-being (anxiety and depression symptoms) in breast cancer patients?

	- (2) Which is the best way to develop avatar-based protocols regarding breast cancer patients?
Eligibility criteria	Inclusion criteria:<br>(1) Primary research studies (journal articles) involving avatar-based strategies to enhance QoL and psychological well-being (anxiety and depressive symptoms) in breast cancer (BC) patients.<br>(2) Published in English or Spanish language between 2012 and 2022.<br>Exclusion criteria:<br>(1) Studies including the use of avatar-based strategies which did not involve BC patients, or they did not measure QoL or psychological well-being (anxiety and depressive symptoms).<br>(2) Protocols with unpublished results, narratives reviews, no journal articles (conference Proceeding, book chapters or Thesis).<br>(3) Other language than Spanish or English.
Information sources	A systematic review was conducted, based on the scientific literature published in EBSCO, Ovid, PubMed, Scopus and Web of Science, throughout the last decade (2012-2022), was carried out in November 2022.
Search strategy	Search strategy for all the database search:<br>Filters: English and Spanish language; year of publication 2012-2022.<br>Conducted in November 2022.<br>The results were extracted with the keywords: “avatar” AND “cancer”; “avatar” AND “breast cancer”.
Type of included study	Only Journal Articles.
Studied domain	Psycho-Oncology; Digital Health.
Population/Participants	Breast cancer patients.

Data collection and selection process	I.R.-G. and P.A.-P. were responsible for data extraction, data analysis, and interpretation. I.R.-G., P.A.-P., C.M.-V. and E.R. substantially contributed to analysis, data interpretation, and revised the work critically. All authors have read and agreed to the published version of the manuscript. All authors were responsible for critical feedback and final revisions of the manuscript.
Data items for coding	The following data will be extracted from the selected articles: (1) publication year, (2) country, (3) study design, (4) study aim, (5) sample size (and mean participants' age), (6) if all the participants included were breast cancer patients and cancer stage, (7) if patients were randomized, (8) training using Avatar, (9) QoL and well-being measured (yes/no instrument used), (10) useful to improve QoL/ psychological well-being, (11) main advantages/disadvantages, and (12) patients' satisfaction.
Outcomes and prioritization	The primary outcomes were the type of avatar-based related treatment developed, if the improvement in mental health was measured through an empirically validated questionnaire, and if the avatar-based strategy involved was useful to enhance QoL and psychological well-being (anxiety and depressive symptoms) in BC patients. The secondary outcomes were the main advantages and disadvantages of the training provided, as well as the patients' satisfaction levels after using the avatars.
Data synthesis	The PRISMA statement will be followed for data synthesis and a narrative synthesis of the included studies will be performed.
Language	English.
Country	Spain.
Anticipated or actual start date	November 2022.

Anticipated or actual end date	November 2022.

Adopted from:

Booth A, Clarke M, Dooley G, Ghersi D, Moher D, Petticrew M, Stewart L. The nuts and bolts of PROSPERO: an international prospective register of systematic reviews. Syst Rev 2012;1(1):2. PMID:22587842

Moher D, Shamseer L, Clarke M, Ghersi D, Liberati A, Petticrew M, Shekelle P, Stewart LA, PRISMA-P Group. Preferred reporting items for systematic review and meta-analysis protocols (PRISMA-P) 2015 statement. Syst Rev 2015;4(1):1. PMID:25554246

Page MJ, McKenzie JE, Bossuyt PM, Boutron I, Hoffmann TC, Mulrow CD, et al. The PRISMA 2020 statement: an updated guideline for reporting systematic reviews. BMJ 2021;372:n71. doi: 10.1136/bmj.n71

Shamseer L, Moher D, Clarke M, Ghersi D, Liberati A, Petticrew M, Shekelle P, Stewart LA, PRISMA-P Group. Preferred reporting items for systematic review and meta-analysis protocols (PRISMA-P) 2015: elaboration and explanation. BMJ 2015;350(jan02 1):g7647. PMID:25555855
