# Supplementary material for: Avatar-Based Strategies for Breast Cancer Patients: A Systematic Review
Source: Cancers (Basel). 2023 Aug 9;15(16):4031. doi: 10.3390/cancers15164031 (PMC10452070; doi:10.3390/cancers15164031)
Supplement: Supplementary file 1 [file cancers-15-04031-s001.zip › cancers-2501174-supplementary file S2.pdf]

Supplementary file S2: Quality assessment of included studies.

Authors	Type study	Screening Questions		Qualitative studies					Randomized controlled trials					Non-randomized studies					Quantitative descriptive studies					Mixed Methods studies				
		S1	S2	1,1	1.2	1.3	1.4	1.5	2.1	2.2	2.3	2.4	2.5	3.1	3.2	3.3	3.4	3.5	4.1	4.2	4.3	4.4	4.5	5.1	5.2	5.3	5.4	5.5
Dang et al. [61]	Mixed-method	Yes	Yes	Yes	Yes	Yes	Yes	Yes	-	-	-	-	-	-	-	-	-	-	-	-	-	-	-	Yes	Yes	Yes	Yes	Yes
Fu et al. [62]	Mixed-method	Yes	Yes	Yes	Yes	Yes	Yes	Yes	-	-	-	-	-	-	-	-	-	-						Yes	Yes	Yes	Yes	Yes
Triberti et al. [63]	Quantitative	Yes	Yes	-	-	-	-	-	-	-	-	-	-	-	-	-	-	-	Yes	Yes	Yes	Yes	Yes	-	-	-	-	-
