# Supplementary material for: Avatar-Based Strategies for Breast Cancer Patients: A Systematic Review
Source: Cancers (Basel). 2023 Aug 9;15(16):4031. doi: 10.3390/cancers15164031 (PMC10452070; doi:10.3390/cancers15164031)
Supplement: Supplementary file 1 [file cancers-15-04031-s001.zip › cancers-2501174-supplementary file S3.pdf]

Supplementary file S3: Reasons for studies exclusion.

Excluded	Reason for exclusion
Baxter [56]	Not QoL or well-being measured.
De Maria Marchiano et al. [57]	Not QoL or well-being measured.
Durosini et al. [1]	Not QoL or well-being measured.
Fu et al. [58]	Usability and feasibility study related with one of included studies [39].
Putranto et al. [59]	Scoping review which includes only one study involving Avatar-based therapy for children (no BC patients)
Ryu & Price [60]	Not QoL or well-being outcomes provided.
